# Sigma-1 Receptor Engages an Anti-Inflammatory and Antioxidant Feedback Loop Mediated by Peroxiredoxin in Experimental Colitis

**DOI:** 10.3390/antiox9111081

**Published:** 2020-11-04

**Authors:** Nikoletta Almási, Szilvia Török, Zsuzsanna Valkusz, Máté Tajti, Ákos Csonka, Zsolt Murlasits, Anikó Pósa, Csaba Varga, Krisztina Kupai

**Affiliations:** 1 Department of Physiology, Anatomy and Neuroscience, University of Szeged, H-6726 Szeged, Hungary; almasi@expbio.bio.u-szeged.hu (N.A.); tszilvia@bio.u-szeged.hu (S.T.); paniko@bio.u-szeged.hu (A.P.); vacs@bio.u-szeged.hu (C.V.); 2 Department of Medicine, Medical Faculty, Albert Szent-Györgyi Clinical Center, University of Szeged, H-6720 Szeged, Hungary; valkusz.zsuzsanna@med.u-szeged.hu (Z.V.); tajti.mate@med.u-szeged.hu (M.T.); 3 Department of Traumatology, University of Szeged, H-6725 Szeged, Hungary; csonka.akos81@gmail.com; 4 Laboratory Animals Research Center, Qatar University, Doha 2713, Qatar; zmrlsits@me.com; 5 Interdisciplinary Excellence Center, University of Szeged, H-6726 Szeged, Hungary

**Keywords:** antioxidants, Sigma-1 receptor, inflammation, IBD (inflammatory bowel disease), peroxiredoxin

## Abstract

Inflammatory bowel disease (IBD), comprising Crohn’s disease (CD) and ulcerative colitis (UC), is a chronic inflammatory condition of the gastrointestinal tract. Since the treatment of IBD is still an unresolved issue, we designed our study to investigate the effect of a novel therapeutic target, sigma-1 receptor (σ1R), considering its ability to activate antioxidant molecules. As a model, 2,4,6-trinitrobenzenesulfonic acid (TNBS) was used to induce colitis in Wistar–Harlan male rats. To test the beneficial effects of σ1R, animals were treated intracolonically (i.c.): (1) separately with an agonist (fluvoxamine (FLV)), (2) with an antagonist of the receptor (BD1063), or (3) as a co-treatment. Our results showed that FLV significantly decreased the severity of inflammation and increased the body weight of the animals. On the contrary, simultaneous treatment of FLV with BD1063 diminished the beneficial effects of FLV. Furthermore, FLV significantly enhanced the levels of glutathione (GSH) and peroxiredoxin 1 (PRDX1) and caused a significant reduction in 3-nitrotyrosine (3-NT) levels, the effects of which were abolished by co-treatment with BD1063. Taken together, our results suggest that the activation of σ1R in TNBS-induced colitis through FLV may be a promising therapeutic strategy, and its protective effect seems to involve the antioxidant pathway system.

## 1. Introduction

Inflammatory bowel disease (IBD) is a chronic, remitting, and relapsing ailment of the gastrointestinal (GI) tract. Two main forms of IBD are Crohn’s disease (CD) and ulcerative colitis (UC), which are distinguished according to the area and the severity of inflammation in the GI tract. CD causes a transmural serious inflammatory damage, while UC is superficial [1]. The therapy of this disease is well-studied and several treatment options have been developed; however, a simple and effective treatment is yet to be discovered [2].

Even though the pathogenesis of IBD is complicated, several studies have demonstrated that excessive biomolecules, cytokines, chemokines, enzymes, and biochemical pathways are involved in the progression of IBD [3]. Basically, CD is considered as a Th1-driven and UC thought to be a Th2-cell-driven disease [4]; however, the latest research suggests the involvement of Th17 cells in both forms of IBD. It has been shown that Th17 inhibition causes a reduction in inflammation, thus contributing to an attenuation of acute colitis [5]. Furthermore, it is increasingly clear that oxidative stress plays a crucial role in the pathogenesis of IBD and in its relapse stage [6].

Several experimental models [7] and clinical studies [8] indicate that oxidative stress has a role in the development of IBD. Prolonged oxidative stress impairs the mucosal layer, resulting in higher bacterial invasion and contributing to the pathogenesis of IBD [6]. Reactive oxygen species (ROSs) primarily refer to free radicals, such as superoxide (O_2_^•−^), hydroxyl radicals (HO^−^), and reactive non-radical compounds including singlet oxygen (O_2_) [9]. Reactive nitrogen species (RNSs) mainly consist of nitric oxide (NO), nitrogen dioxide (NO_2_), and peroxynitrite (ONOO^−^) [10]. Observing oxidative stress mechanisms, 3-nitrotyrosine (3-NT), a protein modification on tyrosine residues, is considered an effective oxidative stress marker of ONOO^−^ [11]. The endogenous antioxidant system, which can counteract the detrimental effects of ROSs, mainly consists of intracellular enzymatic antioxidants, such as superoxide dismutases (SODs), glutathione peroxidase (GPX), and catalase (CAT), as well as intracellular, non-enzymatic antioxidants, including glutathione (GSH) [12]. GSH is a thiol-containing substance with high antioxidant properties [13]. SOD is one of the primary enzymes that convert O_2_^−^ to H_2_O_2_, while peroxiredoxins (PRDXs) are responsible for further deactivation of H_2_O_2_ to H_2_O and the elimination of peroxynitrite to nitrite [14]. The PRDX enzyme family consists of six isoforms (PRDX1-6) [15]. Of these, PRDX1 [16], -2 [17], -4 [18], and -6 [19] are suggested to be factors in IBD. Since oxidative stress is presumably important in the pathogenesis of IBD, enhancement of antioxidant defense has potential benefits in fighting IBD.

Sigma-1 receptor (σ1R) was discovered in 1976 by Martin et al. [20], who proposed it as an opioid receptor. Now, σ1R is confirmed to be a non-opioid receptor that binds highly diverse ligands [21]. σ1R is located in the mitochondria-associated Endoplasmic reticulum (ER) membrane (MAM) and has an impact on Ca^2+^ homeostasis [22]. In response to cellular stress or in the presence of its agonist, σ1R translocates to the plasma membrane and nuclear membrane and modulates ion channels such as the K^+^ channel, NMDA receptors, and IP_3_ receptors [21]. Interestingly, extremely diverse ligand classes show a high affinity for σ1R, including benzomorphans, antipsychomimetics, antihistamines, antidepressants, and antifungal agents [23]. Therefore, this diversity may cause difficulties in a study of the effects of σ1R, because the various ligands may provoke various side effects. Currently, as an agonist, fluvoxamine (FLV, an antidepressant) [24] and cutamesine dihydrochloride (SA4503) are widely applied [25], while BD1063 [26] and NE-100 are frequently used as σ1R antagonists [21].

σ1R is widely studied in the brain and in neurodegenerative diseases, and is currently being suggested as a novel potential therapeutic target against inflammatory conditions [27]. Furthermore, based on our previous findings, the activation of the receptor through the administration of its agonist FLV produces a protective effect in a chemically induced rat model of IBD [28]. Moreover, σ1R has been shown to protect against ER stress and oxidative stress, suggesting a potential therapeutic target against oxidative stress-related diseases [26].

In our present study, we presumed that the protective effect of σ1R in 2,4,6-trinitrobenzenesulfonic acid (TNBS)-induced colitis may arise from activation of antioxidant signaling pathways, primarily via GSH and the peroxiredoxin system. To the best of our knowledge, this paper is the first to study the connection between σ1R and PRDXs in IBD. Our data indicate that FLV significantly increases levels of GSH and PRDX1 antioxidants, and significantly decreases levels of 3-NT. For further confirmation, our combined treatment shows that the presence of the antagonist, BD1063, abolishes the protection exerted by FLV in the colon.

## 2. Materials and Methods

### 2.1. Drug Preparations

As a model of colitis, 2,4,6-trinitrobenzenesulfonic acid (TNBS) (Sigma-Aldrich, Budapest, Hungary) was prepared in 50% ethanol and distilled water mixture. Fluvoxamine (fluvoxamine maleate, Sigma-Aldrich, Budapest, Hungary) was dissolved in 3% dimethyl sulfoxide (DMSO). BD1063, purchased from Tocris (Bio-Techne R&D Systems Kft., Budapest, Hungary), was dissolved in physiological saline (0.9%). The anesthetic agent thiopental (Tiobarbital Braun, 0.5 g, B. Braun Medical SA, Barcelona, Spain) was dissolved in saline (0.9%).

### 2.2. Experimental Animals for the Induction of Colitis

All experiments were performed in accordance with the standards of the European Community guidelines for the Care and Use of Laboratory Animals, and were approved by the Institutional Ethics Committee (XX./4799/2015, 15 December 2015) at the University of Szeged.

Male Wistar–Harlan rats (225–250 g) were purchased from Toxicoop Ltd. (Dunakeszi, Hungary) and were housed in a room with an acclimatized temperature under 12 h day/night cycles with food and water ad libitum. Animals were randomly divided into three groups: absolute control (no treatment, *n* = 12), 50% EtOH (ethanol enema, *n* = 12), and TNBS (10 mg dissolved in 50% ethanol, *n* = 48). Colitis was assessed following Morris’ method [29]. Briefly, animals fasted overnight and a TNBS enema was administered intracolonically (i.c.) with an 8-cm long polyethylene tube through the anus under mild anesthesia (thiopental, i.p. 40 mg/kg). Next, animals from the TNBS-administered group were divided into six groups (*n* = 6–12/group) and further treated once a day with the following drugs under mild anesthesia (thiopental, i.p. 40 mg/kg): fluvoxamine (FLV, σ1R agonist) i.c. administration at the previously tested effective dose 1 mg/kg dissolved in 3% DMSO); BD1063 (σ1R antagonist) 0.1 mg/kg (dissolved in physiological saline (0.9%)); FLV + BD1063 (combined administration of the two effective doses (FLV 1 mg/kg + BD1063 0.1 mg/kg)); saline (vehicle of BD1063); DMSO (3%, vehicle of FLV). The selection of the doses was done according to our previous findings in the same animal model [28]. Animals fasted for 5 h each day before i.c. treatments, which were performed at the same time each day throughout the experiment. The weights of the animals were monitored on the first and third day of the experiment.

After 72 h of TNBS administration, all animals were euthanized (thiopental, i.p. 100 mg/kg), and the last 8-cm portion of the colon was removed, gently opened, rinsed in physiological saline and photographed for further macroscopic analysis. Finally, the colon segments were frozen in liquid nitrogen, powdered using a porcelain mortar and pestle, and kept at −80 °C until needed for biochemical measurements.

### 2.3. Damage Score and Measurement of the Lesions

The extent of macroscopically apparent inflammation, ulceration, and tissue disruption was analyzed in a randomized manner from the images, using a proprietary computerized planimetry software that was developed in our laboratory (Stat_2_1_1, Szeged, Hungary) and was based on planimetrics. The area of macroscopically visible mucosal damage was calculated and expressed as a percentage of the total studied 8-cm colonic segment.

### 2.4. Determination of 3-NT, PRDX1, -2, -4, and -6 Levels in the Colon by ELISA

To determine the tissue levels of 3-NT, PRDX1, -2, -4, and -6 in the colon, we used double-antibody sandwich ELISA kits. The 3-NT kit was purchased from Bioassay Technology Laboratory (Shanghai, China), and PRDX1, -2, -4, and -6 were purchased from GenAsia Biotech Co., Ltd. (Shanghai, China). Samples were homogenized in Phosphate Buffer Saline (PBS), pH 7.4, through the same homogenization procedure (Benchmark Scientific Handheld homogenizer D1000 (Benchmark Scientific, New Jersey, MA, USA); 2 × 10 s, centrifugation = 3000 rpm, 20 min, 4 °C). The whole sample preparation procedure was done on ice. Parameters were measured according to the manufacturer’s instructions and protocols, and optical densities (ODs) were assayed at λ = 450 nm. Results are expressed in nmol/L (3-NT), pg/g protein (PRDX1, -2, -4, -6).

### 2.5. Determination of SOD Activity in the Colon

SOD activity was measured with a kit purchased from Abcam (Cambridge, UK, ab65354). Samples were homogenized in ice-cold homogenization buffer (0.1M Tris/HCl, 0.5% Triton X-100, 5 mM β-ME, 0.1 mg/mL PMSF, pH 7.4). Homogenization was done using a Benchmark Scientific Handheld homogenizer D1000 (Benchmark Scientific, New Jersey, MA, USA); 2 × 10 sec, centrifugation = 14,000× *g*, 5 min, 4 °C. Supernatants were collected and the enzyme activity in the colon samples was measured according to the manufacturer’s instructions, and ODs were determined at λ = 450 nm. From the OD values, the activity of SOD was calculated by an equation listed in the instructions and below, and expressed in inhibition %. In the equation, A = absorbance of Blank1 = 20 μL dH_2_O + Enzyme working solution; Blank2 = 20 μL sample; Blank3 = 20 μL dH_2_O; Sample well = 20 μL sample.

(1) $$SOD\;Activity\;\left(inhibition\;rate\;\%\right)=\frac{\left(\mathrm{Ablank}1-\mathrm{Ablank}3\right)-\left(Asample-Ablank2\right)\;\times \;100}{\left(\mathrm{Ablank}1-\mathrm{Ablank}3\right)}$$

### 2.6. Determination of the Total GSH in the Colon

In order to measure total glutathione levels in the colon, samples were homogenized in 0.25 M sucrose, 20 mM Tris, and 1 mM dithiothreitol (DTT), and centrifuged at 15,000× *g* for 30 min at 4 °C. Supernatants were collected and incubated in a mixture of 0.1 M CaCl_2_, 0.25 M sucrose, 20 mM Tris, and 1 mM DTT at 0 °C for 30 min. After incubation, centrifugation was done at 21,450× *g* for 60 min at 4 °C and the cytosolic fraction was used for further analyses. A solution of 125 mM Na phosphate and 6.0 mM EDTA was used as a diluent buffer for the stock solution of glutathione (GSH), glutathione reductase, 5,5′-dithio-bis-2-nitrobenzoic acid (DTNB) and β-nicotinamide adenine dinucleotide phosphate (β-NADPH). A total volume of 40 µL of each blank, standard, or colon sample, and an equal amount of DTNB stock solution (20 µL) and β-NADPH (140 µL) were added to each well, and then incubated for 5 min at 25 °C. To initiate the reaction, 10 µL of glutathione reductase was added and the absorbance was measured at 405 nm in a microplate reader after 10 min from the initiation. In the spectrophotometric assay for total GSH, GSH was sequentially oxidized by DTNB and reduced by NADPH in the presence of glutathione reductase. Total glutathione values were expressed as nmol/mg protein.

### 2.7. Protein Determination

Protein concentration was measured by the Bradford method. Aliquots of 20 μL of the diluted samples (30× or 40× with distilled water) were taken and mixed with 980 µL distilled water. To each sample 200 μL of Bradford reagent was added, then samples were gently mixed and incubated for 10 min. Spectrophotometric measurements were taken at 595 nm and compared to a bovine serum albumin standard.

### 2.8. Data Representation and Statistical Analysis

All data are presented as mean ± SEM. Statistical analysis was performed using one-way ANOVA followed by a Holm–Sidak post hoc test (SigmaPlot 12, Systat Software Inc., San Jose, CA, USA) for all measurements. Differences were considered significant when the *p* values were less than 0.05.

## 3. Results

### 3.1. Severity of Inflammation Due to Sigma-1 Receptor Associated Treatments in TNBS-Induced Rat Colitis

**Figure 1:**
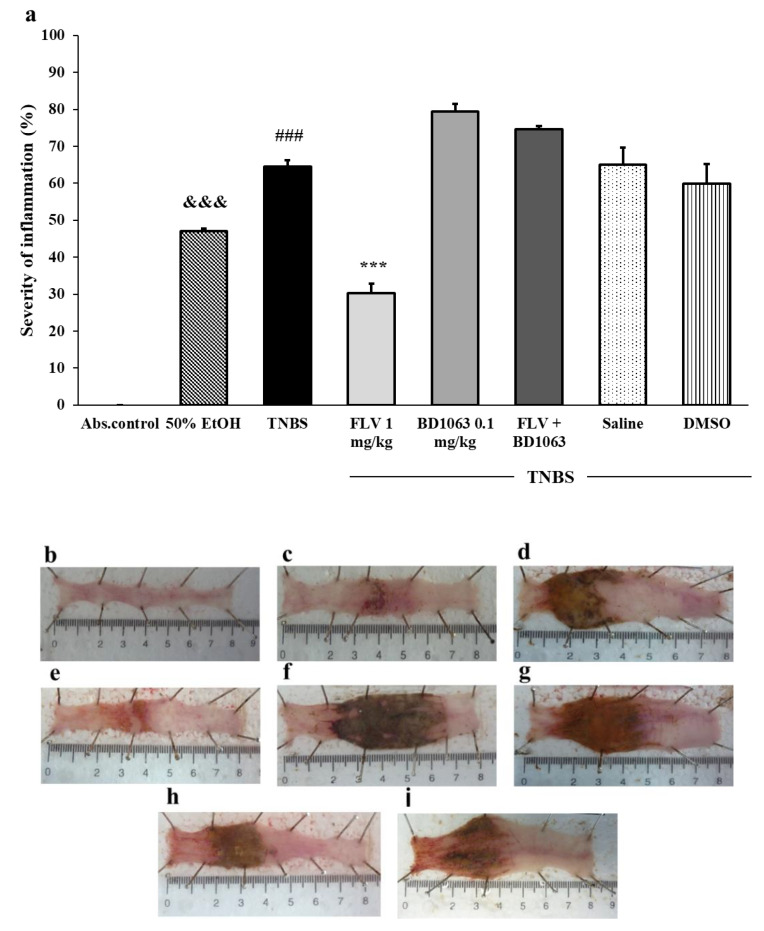
(**a**) Effect of the sigma-1 receptor (σ1R) ligands on the severity of inflammation in 2,4,6-trinitrobenzenesulfonic acid (TNBS)-induced colitis. Representative images of the colonic damage: (**b**) absolute control (no treatment); (**c**) 50% ethanol (EtOH enema); (**d**) TNBS enema; (**e**) TNBS enema + 1 mg/kg fluvoxamine (FLV); (**f**) TNBS enema + 0.1 mg/kg BD1063; (**g**) TNBS enema + 1 mg/kg FLV + 0.1 mg/kg BD1063; (**h**) TNBS enema + Saline; (**i**) TNBS enema + 3% DMSO. Data representation: mean ± SEM; *n* = 5–12/group; *** *p* < 0.001 TNBS vs. TNBS + treatment; ### *p* < 0.001 absolute control vs. TNBS; &&& *p* < 0.001 EtOH vs. TNBS.

As a colitis model, 2,4,6-trinitrobenzenesulfonic acid (TNBS) was dissolved in 50% ethanol (EtOH) and given intracolonically to rats after 12 h fasting. The dissolvent EtOH caused inflammation, ulceration, and necrotic cell death in the colon, but the colonic ulcerations due to TNBS instillation were much more pronounced. In our previous experiment, we determined the effective doses of fluvoxamine (FLV) (1 mg/kg) and BD1063 (0.1 mg/kg) in respect to testing the anti-inflammatory effect of the sigma-1 receptor (σ1R) [28]. Here, we found that the effective dose of the σ1R agonist, FLV significantly reduced the severity of inflammation compared to the TNBS group (30.32% ± 2.46% vs. 64.53% ± 1.68%). Intracolonic administration of BD1063, a σ1R antagonist, exacerbated the colonic inflammation compared to TNBS (79.42% ± 2.46% vs. 64.53% ± 1.68%). To further establish whether this anti-ulcerative effect was a consequence of σ1R activation, we investigated the effect of FLV in the presence of the σ1R antagonist. We found that the presence of BD1063 abolished the anti-inflammatory effect of the agonist, suggesting that the anti-inflammatory action was driven by σ1R activation. We examined the effects of the solvents of FLV (3% DMSO) and BD1063 (physiological saline) and found that solvents of the σ1R ligands did not affect the severity of inflammation compared to the TNBS group (Figure 1).

### 3.2. Body Weight Change of the Animals

**Figure 2:**
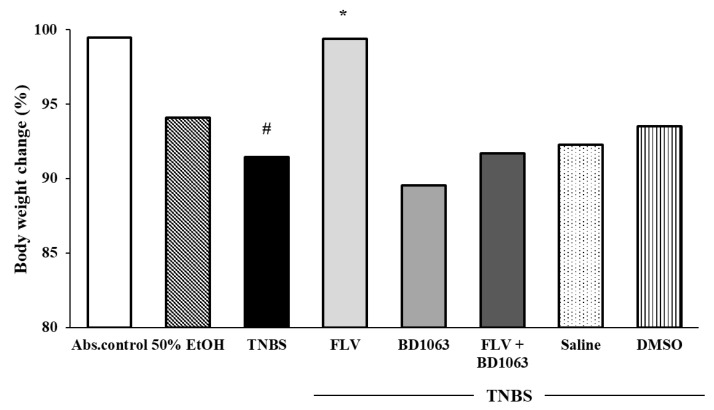
Alterations in the body weight change of the animals throughout our experiment. Body weight change is represented by % between the first and third days of the TNBS procedure. Abs. control (no treatment), 50% EtOH (50% ethanol enema), TNBS (2,4,6-trinitrobenzenesulfonic acid enema), FLV (TNBS enema + 1 mg/kg fluvoxamine (FLV)), BD1063 (TNBS enema + 0.1 mg/kg BD1063), FLV + BD1063 (TNBS enema + 1 mg/kg FLV + 0.1 mg/kg BD1063), Saline (TNBS enema + 0.9% physiological saline), DMSO (TNBS enema + 3% dimethyl sulfoxide). Data representation: mean ± SEM; *n* = 5–12/group; * *p* < 0.01 TNBS vs. TNBS + treatment; # *p* < 0.05 absolute control vs. TNBS.

The body weight change of the animals was monitored throughout our experiments. Due to colonic ulceration and therefore impaired colonic absorption, TNBS instillation was found to cause a significant body weight reduction compared to the weight change of absolute control (91.43% ± 1.77% vs. 99.46% ± 0.51%). Treatment with the σ1R agonist FLV significantly reduced the body weight loss of the animals compared to TNBS (99.40% ± 0.35% vs. 91.43% ± 1.77%). The body weights of the animals treated with FLV were similar to the absolute control group. In our combined treatment, we found that the presence of the antagonist abolished the beneficial effect of FLV according to weight loss. Furthermore, we determined that treatment with the solvents of the σ1R ligands caused no change in body weight loss compared to TNBS alone (Figure 2).

### 3.3. Sigma-1 Receptor Agonist FLV Decreased the Levels of 3-NT in TNBS Colitis

**Figure 3:**
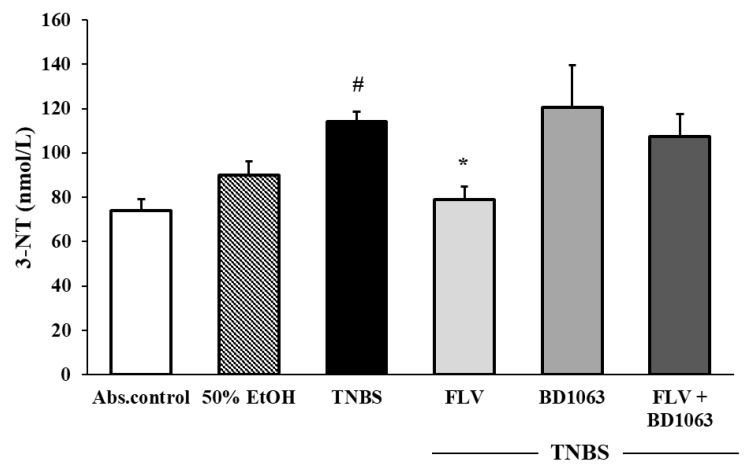
Effects of the sigma-1 receptor on the levels of 3-nitrotyrosin (3-NT) in the colon. Abs. control (no treatment), 50% EtOH (50% ethanol enema), TNBS (2,4,6-trinitrobenzenesulfonic acid enema), FLV (TNBS enema + 1 mg/kg fluvoxamine (FLV)), BD1063 (TNBS enema + 0.1 mg/kg BD1063), FLV + BD1063 (TNBS enema + 1 mg/kg FLV + 0.1 mg/kg BD1063). Data representation: mean ± SEM; *n* = 5–9/group; * *p* < 0.05 TNBS vs. TNBS + treatment; # *p* < 0.05 absolute control vs. TNBS.

As shown in Figure 3, the level of the oxidative stress marker 3-NT was significantly increased in the TNBS group compared to non-treated absolute control (114.24 ± 4.29 vs. 73.93 ± 5.38 nmol/L). FLV treatment significantly decreased 3-NT levels compared to TNBS (79.17 ± 5.5 vs. 114.24 ± 4.29 nmol/L), and BD1063 antagonist slightly enhanced the levels of this parameter. To test the involvement of σ1R in the changes of 3-NT levels, we treated the animals with a combination of the agonist and antagonist and found that the presence of the antagonist abolished the beneficial effect of the agonist on 3-NT levels.

### 3.4. Effects of the Sigma-1 Receptor on the Levels of GSH in TNBS-Induced Colitis

**Figure 4:**
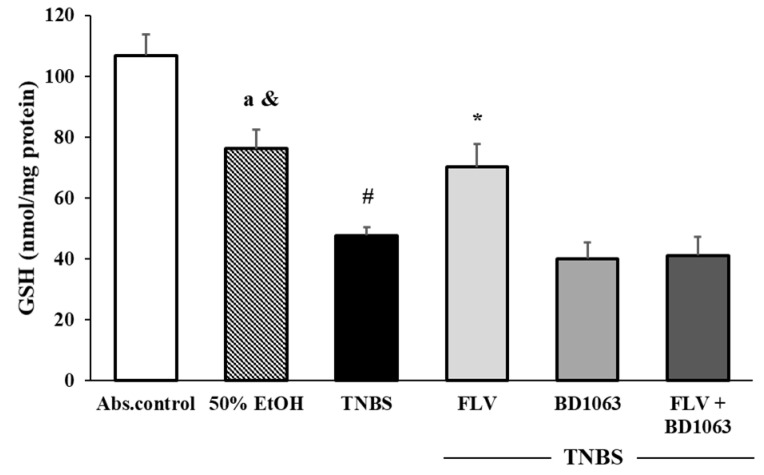
Effects of the activation and antagonism of sigma-1 receptor on the levels of GSH in TNBS-induced colitis. Abs. control (no treatment), 50% EtOH (50% ethanol enema), TNBS (2,4,6-trinitrobenzenesulfonic acid enema), FLV (TNBS enema + 1 mg/kg fluvoxamine (FLV)), BD1063 (TNBS enema + 0.1 mg/kg BD1063), FLV + BD1063 (TNBS enema + 1 mg/kg FLV + 0.1 mg/kg BD1063). Data representation: mean ± SEM; *n* = 5–10/group; * *p* < 0.05 TNBS vs. TNBS + treatment; # *p* < 0.05 absolute control vs. TNBS; & *p* < 0.05 EtOH vs. TNBS; a *p* < 0.05 Abs. control vs. EtOH.

We found that the 50% EtOH enema significantly decreased the levels of GSH compared to absolute control group (76.24 ± 6.35 vs. 106.85 ± 6.94 nmol/mg protein). TNBS significantly reduced the levels of GSH compared to EtOH (47.74 ± 2.68 vs. 76.24 ± 6.35 nmol/mg protein) compared to the absolute control group (47.74 ± 2.68 vs. 106.85 ± 6.94 nmol/mg protein). Compared to the TNBS group, the effective dose of the σ1R agonist FLV significantly elevated the levels of GSH (47.74 ± 2.68 vs. 70.19 ± 7.62 nmol/mg protein). GSH levels were decreased by the administration of BD1063, and also reduced due to the combination of FLV and BD1063 (Figure 4).

### 3.5. Determination of SOD Activity in Sigma-1 Receptor Ligand Treated Rat Colitis

**Figure 5:**
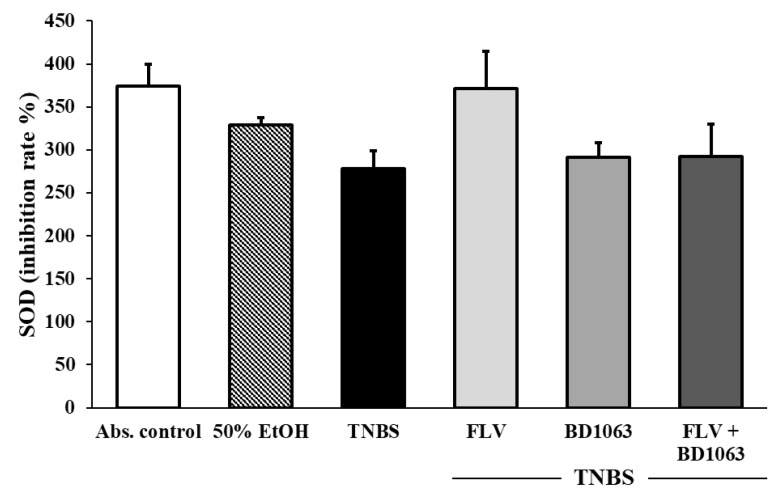
Changes in the activity of the antioxidant superoxide dismutase (SOD) enzyme through the administration of the sigma-1 receptor agonist and antagonist. Abs. control (no treatment), 50% EtOH (50% ethanol enema), TNBS (2,4,6-trinitrobenzenesulfonic acid enema), FLV (TNBS enema + 1 mg/kg fluvoxamine (FLV)), BD1063 (TNBS enema + 0.1 mg/kg BD1063), FLV + BD1063 (TNBS enema + 1 mg/kg FLV + 0.1 mg/kg BD1063). Data representation: mean ± SEM; *n* = 5–11/group.

We determined the activity of the antioxidant SOD enzyme in the colon. The induction of colitis by TNBS decreased the activity of SOD compared to the absolute control group but without significance. Treatment with FLV increased SOD activity, and BD1063 alone or in a combination with the agonist did not change SOD activity compared to the TNBS group, but our results were not statistically significant (Figure 5).

### 3.6. Changes in the Levels of PRDX1, -2, -4 and -6 Due to Sigma-1 Receptor Ligand Administration in Rat Colitis

**Figure 6:**
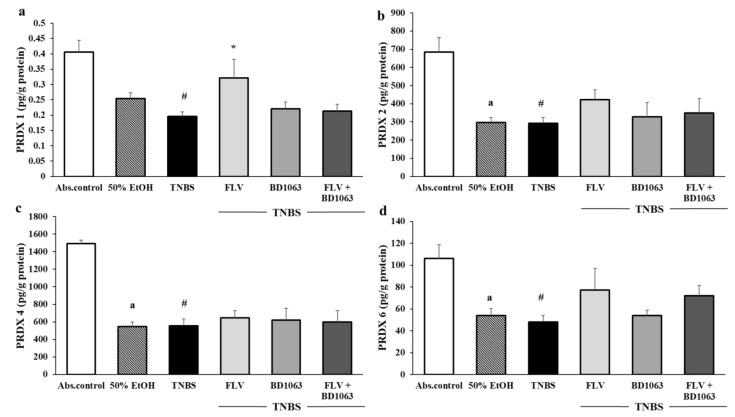
(**a**) The effects of sigma-1 receptor on the levels of peroxiredoxin 1 (PRDX1), (**b**) peroxiredoxin 2 (PRDX2), (**c**) peroxiredoxin 4 (PRDX4), and (**d**) peroxiredoxin 6 (PRDX6) in the colonic tissue. Abs. control (no treatment), 50% EtOH (50% ethanol enema), TNBS (2,4,6-trinitrobenzenesulfonic acid enema), FLV (TNBS enema + 1 mg/kg fluvoxamine (FLV)), BD1063 (TNBS enema + 0.1 mg/kg BD1063), FLV + BD1063 (TNBS enema + 1 mg/kg FLV + 0.1 mg/kg BD1063). Data representation: mean ± SEM; n = 5–12/group; * *p* < 0.05 TNBS vs. TNBS + treatment; # *p* < 0.05 absolute control vs. TNBS; a *p* < 0.05 Abs. control vs. EtOH.

As shown in Figure 6, we measured the levels of four isoforms of the peroxiredoxin enzyme family (PRDX1, -2, -4, -6) in the colon. Intracolonically administered EtOH significantly decreased the levels of PRDX2, -4, and -6 compared to the absolute control group (638.69 ± 79.75 vs. 297.15 ± 27.62; 1494.18 ± 39.69 vs. 545.66 ± 53.03; 106.12 ± 12.61 vs. 53.88 ± 6.33 pg/g protein). TNBS administration significantly decreased the levels of all of the measured PRDX isoforms (0.4 ± 0.03 vs. 0.19 ± 0.01; 638.69 ± 79.75 vs. 292.08 ± 31.7; 1494.18 ± 39.69 vs. 554.57 ± 79.86; 106.12 ± 12.61 vs. 48.02 5.79 pg/g protein); however, daily treatment with FLV significantly increased only the levels of PRDX1 compared to the TNBS group (0.19 ± 0.01 vs. 0.33 ± 0.05 pg/g protein). Furthermore, FLV increased the levels of PRDX2 and PRDX6 without statistical significance, but did not affect PRDX4 compared to TNBS. BD1063 had no impact on the levels of PRDXs in the colon compared to TNBS and, in the case of PRDX1, our combined treatment with the agonist and antagonist showed that the presence of BD1063 abolished the beneficial effect of FLV. This suggests that σ1R has a role in regulating the levels of PRDX1 and possibly modulates PRDX2 and PRDX6.

## 4. Discussion

This study suggests that σ1R activation through the administration of an agonist is protective in a chemically induced rat model of IBD, and the protective effect partially relies on the improvement of the antioxidant-oxidative stress balance. These findings are in line with the previous study of our group [28], which suggest an anti-inflammatory action of σ1R activation by FLV. Our current results show that intracolonically administered FLV, a σ1R agonist, decreases the levels of 3-NT and significantly increases the levels of GSH and PRDX1 antioxidants. To further confirm our findings, BD1063 was used as an σ1R antagonist both alone and in a combined treatment with the agonist. Our data indicate that the presence of BD1063 abolished the beneficial effect of FLV in the case of 3-NT, GSH, and PRDX1, suggesting an interaction with σ1R.

It has been well established that the activation of σ1R protects against ER stress and oxidative stress [30,31]. Administration of σ1R agonists induces the translocation of the receptor and the upregulation of the σ1R itself. Fluvoxamine is one of the frequently used σ1R agonists, and it has recently been reported by our laboratory that locally administered FLV clearly upregulates σ1R expression and efficiently alleviates TNBS-induced colitis [28]. FLV is an SSRI, and it presumably has antioxidant effects through inhibition of the CYP1A2 enzyme [32]; thus, we applied a combined treatment of FLV with a widely used σ1R antagonist BD1063, and analyzed whether the protective function of FLV was affected by the presence of the antagonist.

TNBS-induced acute colitis was developed by Morris et al. [29], and since then has been an extensively used experimental model to burden the pathogenesis of IBD and reveal more effective therapeutic targets [33]. TNBS mimics a Th1/Th17 cell-characterized acute inflammation with a phenotype similar to Crohn’s disease. According to Morris et al., to reach the appropriate ulcer forming feature of TNBS, EtOH is essential. In their study, they found that the administration of EtOH alone or TNBS dissolved in saline caused a less severe superficial inflammation of the colon. On the contrary, TNBS dissolved in EtOH caused a very severe transmural inflammation that was more similar to CD. After intracolonic induction of the haptenating agent, animals suffer from body weight loss that is consistent with malabsorption due to developed ulcers. In our current study, we found that activation of the σ1R, through the administration of FLV, significantly decreased inflammation in rats suffering from TNBS-induced colitis. In addition, this protection was abolished by the co-treatment of FLV with BD1063. Body weight loss was improved as a result of FLV treatment compared to TNBS alone and to combined treatment. Interestingly, Wieczorek et al. [34] found in Zucker rats that intraperitoneal-administered FLV significantly decreased the body weight of the animals without affecting food intake. Thus, we suggest that locally administered FLV to the inflammatory site may act as a healing substance for ulcers through activation of σ1R, which seemed to contribute to an improvement of animal body weight by restoring absorption in our case.

As an oxidative stress marker, 3-NT was found in our study to increase as a result of TNBS administration. Pal et al. [26] found in σ1R KO mice that oxidative stress and ROS generation tend to increase in the livers and lungs of KO mice. Furthermore, it was shown in the same experiment that BD1063 treatment in a COS-7 cell line significantly increased ROS levels, while pentazocine attenuated it. Our results are in accordance with this observation, since we detected a reduced 3-NT level due to FLV treatment and an elevated 3-NT level as an effect of the inactivation of σ1R by BD1063.

GSH is a potent antioxidant substance that protects against oxidative stress. Pal et al. [26] found that the oxidized GSSG form of glutathione increased in the liver of σ1R KO mice, suggesting that the decreased availability of σ1R may contribute to elevated oxidative stress, which is consistent with our current findings on FLV-induced GSH elevation and BD1063-induced GSH depletion. In further accordance with our data, Dursun et al. [35] found in an indomethacin-induced rat stomach ulcer model that orally administered FLV in different doses produced anti-ulceration by altering antioxidant parameters, such as GSH, in a dose-dependent manner. Curiously, Weng et al. [36] found in a σ1R KO mice brain that the loss of σ1R seems to activate a compensatory pathway that leads to antioxidant response element (ARE) activation. Conversely, our results indicate that, besides its suggested action in the central nervous system, σ1R may activate ARE in peripheral tissues. In accordance with this notion, Pal et al. [26] also found that σ1R KO COS-7 cells showed higher ARE activation and SOD1 expression after σ1R transfection. Further elevations in ARE and SOD1 mRNA levels were found after pentazocine treatment, along with a decrease due to the administration of σ1R antagonists such as haloperidol. In our study, we found a marked increase in SOD activity due to FLV treatment in agreement with Elsaed et al. [37], who reported the same alteration in a stress-induced peptic ulcer disease (SPUD) rat model. They suggested that orally administered FLV increased SOD expression and, similar to our results, they also found an attenuation in stomach ulcers due to FLV.

Peroxiredoxins are highly conserved peroxidases with important roles in the elimination, sensation, and regulation of available H_2_O_2_. In mammals, six isoforms are expressed (PRDX1-6), and PRDXs are suggested as potential targets in IBD therapy [16]. According to Zhang et al. [38], PRDX1 decreased significantly in rat dorsal root ganglia (DRG) due to TNBS administration, which is consistent with our current findings. Furthermore, Hsieh et al. [39] found in the colon of severe UC patients that PRDX1 and PRDX2 were downregulated. Additionally, Horie et al. [16] suggested PRDX1 to be a marker of active UC state and UC-associated carcinogenesis. In PRDX4 KO mice, Pfeuffer et al. [18] found a more serious inflammatory reaction to orally administered dextran sodium sulfate (DSS), while interestingly Melhem et al. [19] found that PRDX6 KO mice showed less severe ulceration and a lower rate of inflammation after DSS administration. According to our results, TNBS significantly decreased all four of the measured PRDX isoforms; however, only PRDX1, -2, and -6 seem to have been affected by σ1R alterations. We found that FLV significantly increased the levels of PRDX1 and increased PRDX2 and -6 isoforms without significance compared to TNBS, the alterations of which were abolished by the co-treatment with BD1063. To the best of our knowledge, only one published manuscript [26] has dealt with the possible interaction of σ1R with PRDXs. It was reported that σ1R knockout mice showed an elevated level of PRDX6, which does not support our current findings completely, since we found a decreased PRDX6 level due to BD1063 administration, and a marked elevation as a result of the presence of FLV. We presume that this discrepancy may arise from the use of different species.

## 5. Conclusions

Based on the current data, we suggest that the protective, antioxidant effect of σ1R, due to FLV treatment, seems to rely on the attenuation of oxidative stress. Moreover, it was shown that FLV treatment is able to activate PRDX1 isoform and GSH. This restored antioxidant balance may contribute to the anti-inflammatory and protective effect of σ1R activation against TNBS-induced colitis. Our results may offer a new therapeutic target against this serious health issue and shed light on the function and role of this enigmatic receptor.
